# A Journey to the West: The Ancient Dispersal of Rice Out of East Asia

**DOI:** 10.1186/s12284-021-00518-4

**Published:** 2021-09-25

**Authors:** Robert N. Spengler, Sören Stark, Xinying Zhou, Daniel Fuks, Li Tang, Basira Mir-Makhamad, Rasmus Bjørn, Hongen Jiang, Luca M. Olivieri, Alisher Begmatov, Nicole Boivin

**Affiliations:** 1 Department of Archaeology, Max Planck Institute for the Science of Human History, Jena, Thuringia Germany; 2 Institute for the Study of the Ancient World, New York University, New York City, NY USA; 3 Key Laboratory of Vertebrate Evolution and Human Origins, Institute of Vertebrate Paleontology and Paleoanthropology, Chinese Academy of Sciences, Beijing, China; 4 Department of Archaeology and Anthropology, University of Chinese Academy of Sciences, Beijing, China; 5 Center for Excellence in Life and Paleoenvironment, Chinese Academy of Sciences, Beijing, China; 6 McDonald Institute for Archaeological Research, University of Cambridge, Department of Archaeology, Cambridge, UK; 7 Martin (Szusz) Department of Land of Israel Studies and Archaeology, Bar-Ilan University, Ramat Gan, Israel; 8 Dipartimento di Studi sull’Asia e sull’Africa Mediterranea, Università Ca’ Foscari Venezia, Venice, Italy; 9 ISMEO - International Association for Mediterranean and Oriental Studies, Rome, Italy; 10 Berlin-Brandenburg Academy of Sciences and Humanities, Turfanforschung, Berlin, Germany; 11 Department of Anthropology, Smithsonian Institution, National Museum of Natural History, Washington, DC USA; 12 School of Social Science, The University of Queensland, Brisbane, Australia; 13 Department of Anthropology and Archaeology, University of Calgary, Calgary, Canada

**Keywords:** Rice, Paddy farming, West Asia, Archaeobotany, Agricultural intensification, Crop exchange

## Abstract

Rice is one of the most culturally valued and widely grown crops in the world today, and extensive research over the past decade has clarified much of the narrative of its domestication and early spread across East and South Asia. However, the timing and routes of its dispersal into West Asia and Europe, through which rice eventually became an important ingredient in global cuisines, has remained less clear. In this article, we discuss the piecemeal, but growing, archaeobotanical data for rice in West Asia. We also integrate written sources, linguistic data, and ethnohistoric analogies, in order to better understand the adoption of rice outside its regions of origin. The human-mediated westward spread of rice proceeded gradually, while its social standing and culinary uses repeatedly changing over time and place. Rice was present in West Asia and Europe by the tail end of the first millennium BC, but did not become a significant crop in West Asia until the past few centuries. Complementary historical, linguistic, and archaeobotanical data illustrate two separate and roughly contemporaneous routes of westward dispersal, one along the South Asian coast and the other through Silk Road trade. By better understanding the adoption of this water-demanding crop in the arid regions of West Asia, we explore an important chapter in human adaptation and agricultural decision making.

## Introduction

Asian rice (*Oryza sativa*) is currently the second most-consumed food crop by humans, after wheat (*Triticum aestivum*), and a major global primary commodity (FAO 2020). The intensification of rice cultivation, along with maize (*Zea mays*) and wheat, fueled major demographic transition of the past ten millennia (Ammerman and Cavalli-Sforza 1984; Bocquet-Appel 2011; Gowdy and Krall 2014; Omrak et al. 2016; Brace et al. 2019; Soares et al. 2019). Rice’s global economic importance is perhaps all the more impressive considering that its westward spread through Eurasia began several millennia after wheat’s eastward diffusion from southwest Asia, the latter process spanning from roughly 6500 to 2500 BC (Zohary 1998). Archaeobotanists have elucidated much of the path towards initial domestication, dispersal, and intensification of cultivation. However, the timing and specific routes of dispersal of rice out of East Asia remain elusive, due in part to a dearth of data from early historical periods in West Asia. In this paper, we trace what appears to be two roughly contemporaneous and parallel westward dispersals. One across the Silk Road through the southern mountains and arid Central Asia, and another through South Asian nautical trade, i.e. the Sea Links or maritime routes across the Indian Ocean along the Red Sea and Mediterranean, to Europe. Rice is a water-demanding crop and its spread into West Asia has been argued to coincide with greater collective investment in irrigation and shifting culinary practices (Watson 1983). By tracing the route of dispersal westward for this key global staple, we seek to better understand the processes that led to the modern cultural, demographic, and economic world.

While there have been many attempts to synthesize the early narrative of rice domestication and dispersal, notably Fuller (2011a) in the journal *Rice*, all of these end at the deserts or mountains of West Asia. One of the most fascinating and least studied chapters in the story of rice starts when it leaves its East Asian origins and spreads into arid West Asia and through the Red and Mediterranean Seas (Nesbitt et al. 2010; Muthukumaran 2014; Van Der Veen and Morales 2015; Reed and Leleković 2019). With increasing discovery of archaeobotanical rice remains from West Asia and Europe, it is worth exploring what we can say about the social implications of its spread. Rice is an inseparable part of cuisines from Xinjiang to Azerbaijan; it plays an important role in the economy in arid regions of the Arabian Peninsula, North Africa, and across the Iranian Plateau. Likewise, versions of *pilaf* (*palov, palau, plov*, etc.), are served across West Asia, Eastern Europe, and North Africa today (Nesbitt et al. 2010; Muthukumaran 2014). Pilaf is a symbol of national identity for people from Uzbekistan and Afghanistan to Morocco; while there are many regional derivatives, the core ingredient is always rice. Summer paddy rice cultivation is still practiced in some of the most arid regions of West Asia, notably in Mazandaran and Gilan, Iran, as well as Zanjan, Golestan, Khuzestan, Esfahan, Fars, and Khorasan, with valuable water in oases being diverted towards rice rather than arid-land-adapted crops (Nesbitt et al. 2010). Understanding how and when rice traversed two continents, informs us about adaptive strategies, culinary choices, and globalization in the ancient world.

## Domestication

Despite major scientific advances in the past few years, rice domestication remains a topic of debate. We present a brief summary of current views here, but more detailed summaries have been published elsewhere. Two clear genetic and phenotypic lineages of rice exist, *O. sativa* ssp. *japonica* in East Asia and *O. sativa* ssp. *indica* in South Asia (Civán et al. 2015; Castillo et al. 2016), evolving from two different progenitors, *O. nivara* (*indica*) and *O. rufipogon* (*japonica*) (Kawakami et al. 2007). However, the origin story is complicated by the fact that several alleles associated with domestication are shared in common between the two lineages, a realization that has led scholars to suggest that domestication may be linked to hybridization (Kovach et al. 2007). Current consensus among scholars suggests that two lineages were independently brought under cultivation, one in northern India and one in eastern China (Fuller et al. 2010; Fuller 2011a, b; Gross and Zhao 2014; Castillo et al. 2016). In this model, the traits of domestication, notably a tougher rachis (the *sh4* allele), evolved only once in a cultivated population in East Asia, and humans dispersed the domesticated morphotype across the continent more than five millennia ago. Subsequent studies have also suggested hybridization with repeated back crossing (McNally et al. 2009). When and how this original hybridization occurred remains poorly understood and requires further research.

### East Asia

The origins of East Asian rice are more clearly understood than the South Asian lineage, benefiting from an immense wave of archaeobotanical investigation. Rice was likely collected from the wild by seed foragers for millennia before it was cultivated (Gross and Zhao 2014). Evidence has been presented for more targeted rice foraging by 8000 years ago along the lower Yangtze River (Zhao 2011). Tianloushan and Hemudu in the Yangtze floodplain have provided some of the earliest evidence for cultivation and illustrate that rice was still a minor component in a more complex foraging strategy that included fruits and nuts (Fuller et al. 2009). The increased focus on rice in the economy only took place within the past five thousand years. Rice cultivation spread into the middle and upper Yangtze region by 6000 BC (Zhang et al. 2012; Jin et al. 2014; He et al. 2017; Lu 2017). Fuller et al. (2007) argue that non-shattering spikelets of rice were not fixed into the population until after 5000 BC, with full populations of domesticated rachises reported by 4000 BC (Fuller et al. 2009, 2014). Supporting this conclusion, rice spikelet bases from Kuahuqiao (6000–5400 BC) were primarily of a wild morphotype (Zheng et al. 2004). At the site of Huxi, non-shattering spikelet bases have been recorded, dating to 6500 BC, and possibly represent the oldest evidence for a beginning of the domestication (Zheng et al. 2016). These close analyses of diachronic phenotypic change in rachis structure appear to call earlier claims of domestication based on phytoliths into question (e.g. Liu 2004; Jiang and Liu 2006). Despite the seemingly clear sequence for morphological change through time, subsequent phytolith studies have also claimed earlier dates for rice domestication and dispersal (Zhang et al. 2010; Zuo et al. 2017).

Archaeobotanists have argued for a rapid northward and westward dispersal of rice into the Guanzhong Plain and the Hehuang region of Gansu (Zhang et al. 2010, 2012; Jin et al. 2014). Current evidence seems to imply a slightly later dispersal south and west (Chen et al. 2020; He et al. 2017). Deng et al. (2018) recently claimed to identify domesticated rice bulliform phytoliths in eastern Taiwan at Chaolaiqiao from 4200 years ago. Rice cultivation appears to have made it to the Sichuan Plains at Baodun and Guiyanqiao by more than 4000 years ago (d’Alpoim Guedes et al. 2013) and into Yunnan at Baiyangcun by 2640 BC (Dal Martello et al. 2018). The lack of early archaeobotanical evidence for rice in the Hexi Corridor or northern Central Asia, has led scholars to suggest that ecological constraints would have hampered a northern dispersal (Chen et al. 2020). Increasingly, scholars are accepting a demic expansion model, suggesting that grain surplus allowed for the growth and spread of farming populations along with cultivation techniques across Asia (Fuller 2011b; Cobo et al. 2019).

### South Asia

Indian rice domestication has been a topic of extensive inquiry over the last two decades (Fuller and Madella 2002; Saraswat 2005; Fuller 2005, 2006, 2011a; Tewari et al. 2008; Fuller et al. 2010; Bates et al. 2016, 2017a; Petrie and Bates 2017). As in East Asia, phytoliths have been used in South Asia to argue for rice cultivation and domestication thousands of years earlier than evidenced by archaeobotanical remains. Notably, phytoliths from a 2.8 m-deep lake-profile trench at Lahuradewa in Uttar Pardesh have been used to argue for wild rice harvesting by 8300 BC and full cultivation by 5000 BC (Saxena et al. 2006); these early dates have been repeatedly referenced (Saraswat 2005; Saxena et al. 2006; Pokharia et al. 2017). Charred rice grains were found in the earliest phase of Lahuradewa, site phase 1A (ca. 6400 BC), and using morphometrics Tewari et al. (2008) argued that these grains were domesticated. Many scholars are highly critical of these early rice dates, notably regarding their domesticated status (Fuller 2002; Harvey et al. 2005; Possehl 1999). Scholars have questioned the reliability of the domestication status of rice phytoliths at other sites in South Asia as well (Fujiwara et al. 1992; Fujiwara 1993; Fuller and Madella 2002; Bates et al. 2016, Bates and Petrie 2016). Deng et al. (2018) illustrate how widely varying in morphology rice bulliform phytoliths can be, even within a single population.

More secure evidence for rice consumption and harvesting, either wild or cultivated, comes from several second and late third millennium BC sites (Harvey and Fuller 2005; Saraswat 2005; Fuller 2011a; Bates et al. 2016, 2017a; Stevens et al. 2016; Petrie and Bates 2017). The first evidence for the increase and concentration of human populations and the formation of villages in the Ganges, Punjab, Harayana, and Swat dates to roughly 2500 BC and coincides with archaeobotancial rice remains (Costantini 1987; Saraswat and Pokharia 2003; Fuller 2006; Harvey et al. 2005; Fuller 2011b; Silva et al. 2015). Rice cultivation was well-established across the Ganges region by the mid-third millennium BC and much of India and southeast Asia by the mid-second millennium BC (Fuller 2002, 2005, 2006, 2011a; Fuller and Madella 2002; Saraswat 2005; Tewari et al. 2008; Fuller and Qin 2009; Fuller et al. 2010; Madella 2014; Silva et al. 2018). Its significance as a summer crop in seasonal rotation systems appears to have been geographically variable, and scholars have noted that farming systems across India were dynamic through time and space (Petrie and Bates 2017; Bates et al. 2017b).

## Himalayan Plateau

Considerable focus has been placed on a rice dispersal route across southern South Asia (Fuller 2011a, b; Castillo et al. 2016; Stevens et al. 2016), although Stevens et al. (2016; Fuller 2011a) cautiously entertain a northerly route, noting there is no genetic signature of *japonica* in the greatest rice-growing regions of Myanmar, Assam, and Bengal. They state, “for rice, as with Chinese millet entry from the northwest is suggested by the admittedly patchy data, but we would stress the need for increased archaeobotanical sampling around both northwest and northeast South Asia and throughout the Himalayas” (Stevens et al. 2016:1550). In between the northern and southern routes, many scholars have proposed that East Asian rice first dispersed into northwestern India through mountain passes along rich river valleys (Vaughan et al. 2008; Vidale et al. 2011; Huang et al. 2012; Chen et al. 2020), such as Kashmir, where early rice impressions in ceramics are reported (Costantini 1987).

East Asian style harvesting knives, jade and other stone objects, and tripod ceramic vessels appear in this region in the third and second millennia BC; these artifacts are emblematic of cultural dispersal from the east (Stacul 1976, 1993; Sharif and Thapar 1992; Fairservis 1975; Han 2012; Coningham and Young 2015). This “Chinese Horizon” may mark the introduction of *japonica* rice along with peaches (*Prunus persica*) and apricots (*P. armeniaca*) from East Asia (Fuller 2006; Fuller et al. 2010; Boivin et al. 2012; Stevens et al. 2016). Human aDNA suggests that the southern Himalaya served as a genetic barrier to low-elevation populations, which were not physiologically adapted to hypoxia and other ecological constraints (Jeong et al. 2016). Recent genetic studies also suggest long periods of population continuity in rich mountain valleys, notably the Swat Valley (Narasimhan et al. 2019). Despite a lack of evidence for human genetic movements through the southern Himalayan valleys, there is considerable evidence for the movement of goods and crops (Spengler et al. 2020). Discussions of crop dispersal in this region are often closely linked to the concept of a “Northern Neolithic”, suggesting that a unique developmental trajectory was followed by farmers in Swat and Kashmir (Betts et al. 2019). The presence of rice in these valleys by the late second or first millennium BC means that people living in areas in the mountain foothills—in some cases where rice cannot grow today—were responsible for transporting viable seeds over thousands of kilometers (Fuller 2011a). Recent archaeobotanical research on the Himalayan Plateau has led to the identification of early rice remains at high elevations, above its growing zone, suggesting that small-quantities of rice might have been transported over short distances. The site of Kaerdong (also referred to as Kyung-lung Mesa; 4300 masl) is thought to have had a relationship with the Zhangzhung (500 BC–AD 625), a pre-Buddhist kingdom in the western Himalayas (Aldenderfer and Olsen 2008). Abundant archaeobotanical remains were collected from test trenches in 2004 and 2013. A single rice grain was recently recovered in association with a barley grain that was directly dated to between AD 455 and 700. Surprisingly, the researchers report nine rice spikelet bases from different layers, which may suggest that rice was consumed by higher status individuals and the grains were carried to higher elevations un-threshed (Song et al. 2018). Rice grains were also recovered from the nearby large-scale settlement of Zebang (4100 masl), which dates to roughly the same period (Song et al. 2018).

Rice was reported at Semthan in Kashmir dating between 1500 and 500 BC (Lone et al. 1993), and from the later phases at Gufkral and Burzahom also in the Kashmir Valley (ca. 1800–1000 BC; Kajale 1982; Lone et al. 1993). Although, Pokharia et al. (2018) note that there is some question about the exact stratigraphic context of the rice grains at these sites. The dense archaeological deposits in the valleys of Kashmir, Kapisa, and Swat in the western Himalayan-Hindu Kush region of India, Pakistan and Afghanistan illustrate that people were concentrating in these ecologically rich orographic zones and that the passes served as corridors of exchange from the third millennium BC until today (Bandey 2009; Han 2012; Coningham and Young 2015; Vidale et al. 2015; Olivieri 2018; Olivieri et al. 2019). Archaeologists have argued for an established farming system in the Swat Valley at least by the beginning of the second millennium BC, based on archaeological data and material culture and expansion of settlement size, as well as from inferred data such as the apparent demographic shifts (Vidale et al. 2011). In the second millennium BC geometric rock-art depictions of Swat have been interpreted as field cultivation scenes (Vidale and Olivieri 2002; Vidale et al. 2011).

Agricultural tools have been recovered in Swat notably the so-called ‘perforated knives’ of the Northern Neolithic, as well as saddle-shaped querns (Vidale et al. 2011). Domesticated zebu are also attested, at least since the third millennium BC (Costantini 1987). Since protohistory, and until pre-modern times, the surplus of rice and other cereals from Swat was exported to the major centers of the plain of Gandhara (Olivieri, 2020). An intensive and complex agricultural system was recently identified at the multiphase site of Barikot in the studied period (ca. 1200 BC-50 AD) in the Swat Valley (Olivieri et al. 2019), whereas rice was likely a summer crop in a mixed cropping system (Spengler et al. 2020; Fig. 2h, j). The diversity of different crops in the Barikot assemblage is reflective of the unique cultivation conditions in the valley, allowing warmer-weather crops, characteristic of the Indus, to grow further north. While it is unlikely that rice at Barikot represents the earliest remains in the region, it shows that rice was a well-established part of the farming system in these mountain valleys before the tail end of the second through the first millennia BC.

## West and Central Asia

### Historical Sources

Linguistics can clarify some of the contours of the trajectories of dispersal. The majority of words for rice in Central Asia are, ultimately, borrowed from a form similar to Old Chinese *brêh ‘fine rice’ (cf. Witzel 1995: 102), and shows up in two distinct yet obviously related forms: in the south and east as Vedic *vrīhí*- and Pashto *wriže* (cf. also in the isolate Burushaski *brás* and *bríu*, where the exact origin remains elusive), and in the west as Persian *brinj* and *gurinj* (Mayrhofer 1976: 282). In Ancient Greek *orindas* (‘rice cake’) reflects the western form, and the generic term *oryza* (‘rice grain’) is a borrowing of the eastern variant, which also lies behind English *rice*, Khotanese *rrīysu*, and Yaghnobi *rijan* (Chantraine 1968: 820, 828). The western word also continues in the various Turkic languages that borrowed the Iranian forms (e.g. Turkish *pirinc*, Kazak *kürish*, and Uighur *gurunj*; Bailey 1979: 364). While the Iranian word entered the Tarim Basin from the west (Khotanese), there is a distinct eastern word for rice that appears in local Tocharian languages (Tocharian A and B *klu*) that must derive from another Sinitic word (Old Chinese *[l]ˤuʔ* [unhusked rice]; Peyrot 2018: 254–255), suggesting two distinct entries to eastern Central Asia. Another term for rice, which is prevalent across Central Eurasian languages (mainly Iranian and Turkic), *šālī* specifically refers to unhusked rice. This term is likely borrowed from Sanskrit *śāli*- (Morgenstierne 2003: 78), where it in turn represents a local borrowing (Mayrhofer 1976: 632).

The most frequently cited text associated with the dispersal of rice into West Asia is *The Records of the Great Historian*, the *Shiji* (Sima 1993 [109-91 BC]: Book 123: 3160). Zhang Qian, the first Han ambassador to the ‘Western Regions’, is credited with having reported that rice was cultivated in Dayuan, 大宛, (as well as Parthia and Chaldea, mentioned below), according to most scholars the historical region of Ferghana. There are few written indications of rice in western Central Asia over the following 500 years, but this may be due to the general dearth of written sources. There is a mention of rice fields in Dayuan in the *Jinshu* (Fang 1974 [AD 265-420]: Volume 97: 2542), but some parts of the *Book of Jin* appear to be transmitted from the earlier *Shiji*. Rice does not appear in Avestan texts (although, only a handful of plants are mentioned in those texts; Boyce 1979), nor is it among the grains that Herodotus mentioned growing in Persia (Herodotus 1987 [440 BC]). Likewise, it was stated in the *Weishu* (Wei 2018 [AD 551-554]: Volume 102: 2271) that no rice was grown in Bosi, an account likely gathered by a Northern Wei embassy in AD 436 (Agostini and Stark 2016). This statement is simply repeated in the *Beishi* (Li 2018 [AD 643-659]: Volume 97: 3222) and the *Zhoushu* (Linghu 2018 [AD 636]; Volume 50: 920), suggesting that rice was still rare across southwest Asia. Moreover, rice is not attested in the Aramaic documents from ancient Bactria (modern Turkmenistan; fourth century BC) and in any Bactrian documents (fourth to eight centuries AD), in which wheat, barley, and millet are cited (Naveh and Shaked 2012; Sims-Williams 2012). Rice is also lacking in the economic Sogdian documents from Mugh in the Upper Zerafshan Valley (early eighth century AD), in which various kinds of grains are discussed in great length (Bogolyubov and Smirnova 1963).

In the fifth century AD, the *Weishu* references rice cultivation across several regions of Central Asia, including: (1) Nakhshab (Nuoseboluo 諾色波羅) in the Lower Kashka-Darya (Volume 102: 2273); (2) Kabudhan (Jiabudan 伽不單) in the Bulungur zone of the Samarkand Oasis (Volume 102: 2273); and (3) possibly also in Bukhara. Although, the claim for Bukhara is only indirect, as rice is only mentioned in relation to the small principality of Zaojiazhi (早伽至) west of Bukhara (Niumi 忸密), as we note below, this may be Paykend (Weishu, Volume 102:2273). Records from the Chinese travelers, Xuanzang and Hyecho, recorded local foods and rice seldom appears, but they note that Persians favored bread. The *Hulin* (Tang period; AD 618-907) notes that the Sogdians cooked the dish *Hualuo* (in Middle Chinese this probably sounded somewhat closer to pilaf). The *Weishu* accounts were likely gathered by a Northern Wei envoy to Ferghana and Chach (the area around modern Tashkent) in AD 437 (Yu 2013). Although, as mentioned above, rice is not attested in Sogdian economic documents, historians note two different terms for rice, *βrinj (brync)* and *rīzā* (*ryzʾkh*), in the Sogdian Buddhist and Manichaean texts (Benveniste 1940: 48). The former is a loanword from Middle Persian. The latter is a cognate of a widely spread term in many European languages. In addition, Sogdian historians note a term for a rice field, *ryzʾkh ʾstny* (Benveniste 1940: 48). Rice likely reached Sogdiana from the south (from Bactria, Gandhara, and northern India)—this is suggested by the fact that rice is mentioned in Sogdiana, but not in Chach (Zheshe 者舌), and the southern origin of the etymon—perhaps as a result of Sogdian commerce with northern India, or as a result of political and cultural connections with the regions to the south during the Hunnic period (Kidarite and Hephthalite rule in Sogdiana, Bactria, and Gandhara—see Alram 2016). The different word for rice in the autochthonous Tocharian languages of the Tarim Basin suggests that it is less likely that rice dispersed with Sogdian diaspora communities. As a final note, the state of Zaojiazhi, 早伽至, from the *Weishu*, Chapter 102 (ca. AD 437), supposedly had only a “few agricultural fields” so that “rice and wheat are imported from the neighboring countries”. Arguably, the text refers to Paykend, where rice may have been imported, and where recent archaeobotanical finds of rice are discussed below.

### Archaeobotanical Data

**Fig. 1:**
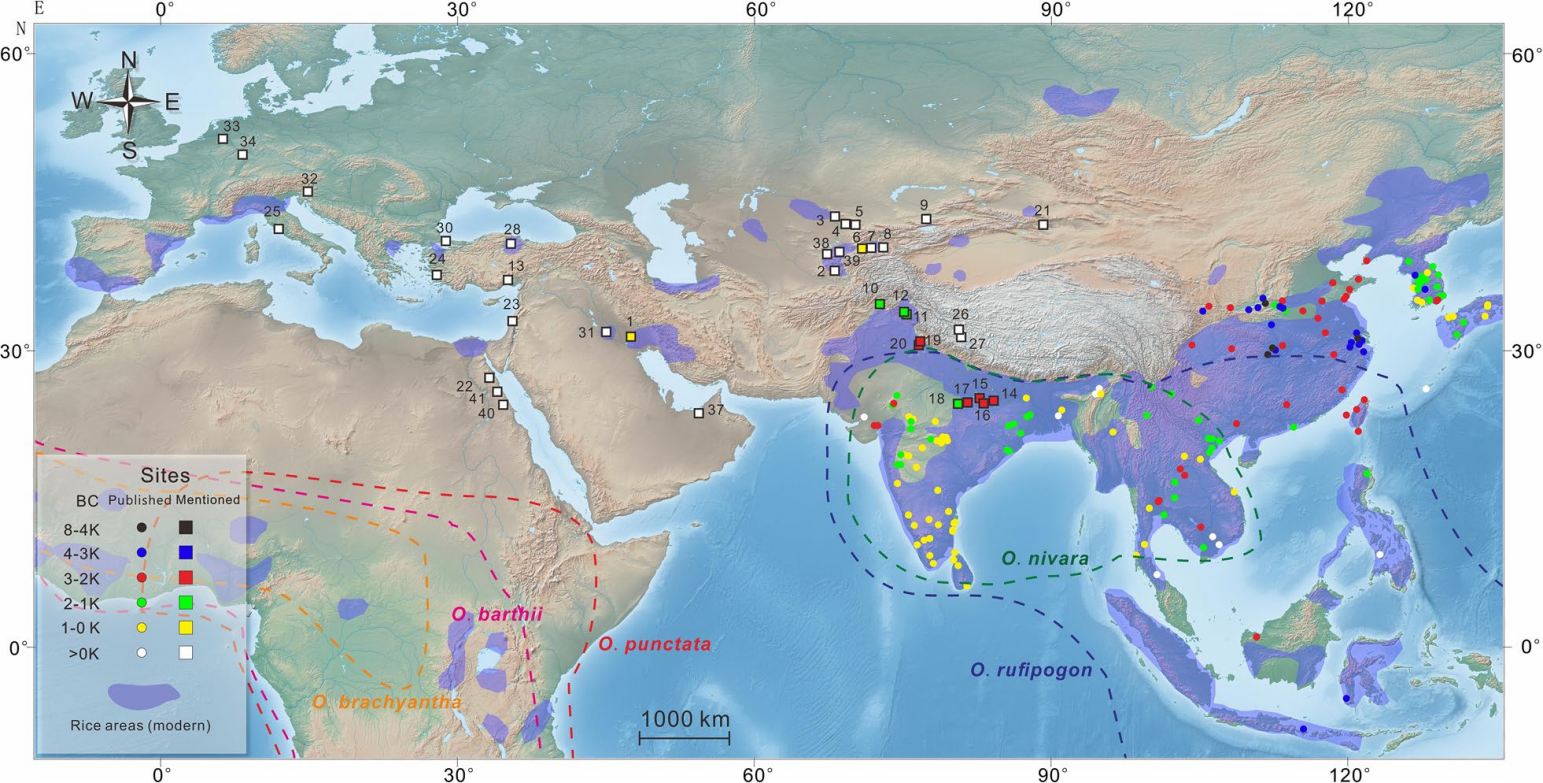
Map illustrating the ancient dispersal of rice; blue shaded areas represent the range of modern rice cultivation as modified from Ray et al. (2012); modern range of wild rice species are indicated by dotted lines; (1) Susa (Strabo 1924 [7 BC-AD 23]; Miller 1981); (2) Khalchayan ca. AD 250 (Chen et al. 2020); (3) Konyr-tobe, seventh century AD (Bashtannik 2008); (4) Karaspan-tobe, fourth to fifth century AD (Bashtannik 2008); (5) Djuvan-tobe, seventh century AD (Bashtannik 2008); (6) Khujand (Sima 1993 [91–109 BC]); (7) Nos 28, 29, and 61, early first millennium AD (Gorbunova 1986); (8) Munchak Tepe, fifth to seventh centuries AD (Gorbunova 1986); (9) Paykend, ca. 1100 AD (Mir-Makhamad et al. in review); (10) Barikot, ca 1200 BC (Spengler et al. 2020); (11) Semthan 1500–500 BC (Lone et al. 1993); (12) Gufkral and Bruzahon, ca. 1800–1000 BC (Kajale 1982); (13) Tarsus (Dioscorides 2000 [AD 64]); (14) Senuwar (ca. 2200–600 BC); (15) Jhusi (ca. 2200–1900 BC); (16) Chopani Mando (third millennium BC); (17) Koldihwa (third millennium BC); (18) Mahagara (early second millennium BC); 19) Bahola (second millennium BC); (20) Masudpur (second millennium BC); (21) Astana Cemetery, AD 304–439 (Chen et al. 2012); (22) Quseir al-Qadim, second century AD (van der Veen and Morales 2015); (23) Mishna (second century AD); (24) Lesbos (Theophrastus 1916 [350–287 BC]); (25) Rome (Apicius 1984 [first century AD]; (Pliny the Elder 1855 [AD 77–79]); Horace (2008 [35 BC]); (26) Kyung-lung Mesa, AD 455–700 (Song et al. 2018); (27) Zebang, first millennium AD (Song et al. 2018); (28) Amasya (Strabo 1924 [7 BC-AD 23]); (29) Agira (Diodorus 1967 [ca. 60 BC]); (30) Istanbul (Anthimus 1996 [AD 500–525]); (31) Babylonian Talmud (sixth century AD); (32) Aelia Mursa, early second century AD (Reed and Leleković 2019); (33) Novaesium, early first century AD (Knörzer 1970); (34) Mogontiacum, late first millennium AD (Zach 2002); (35) Teshik-Kala in Kharasam, between seventh and eighth century AD (Brite et al. 2017); (36) Erkala (Merv) probably from the third century AD (Usmanova 1963); (37) Mleiha in the United Arab Emirates, third century AD) (Dabrowski et al. 2021); (38) Bukhara, ca. 1000 AD, ongoing studies; (39) Afrasiab, ca. 1000 AD, ongoing studies; (40) Berenike, first centuries AD (Cappers 2006); (41) Myos Hormos, first centuries AD (van der Veen 2011)

**Fig. 2:**
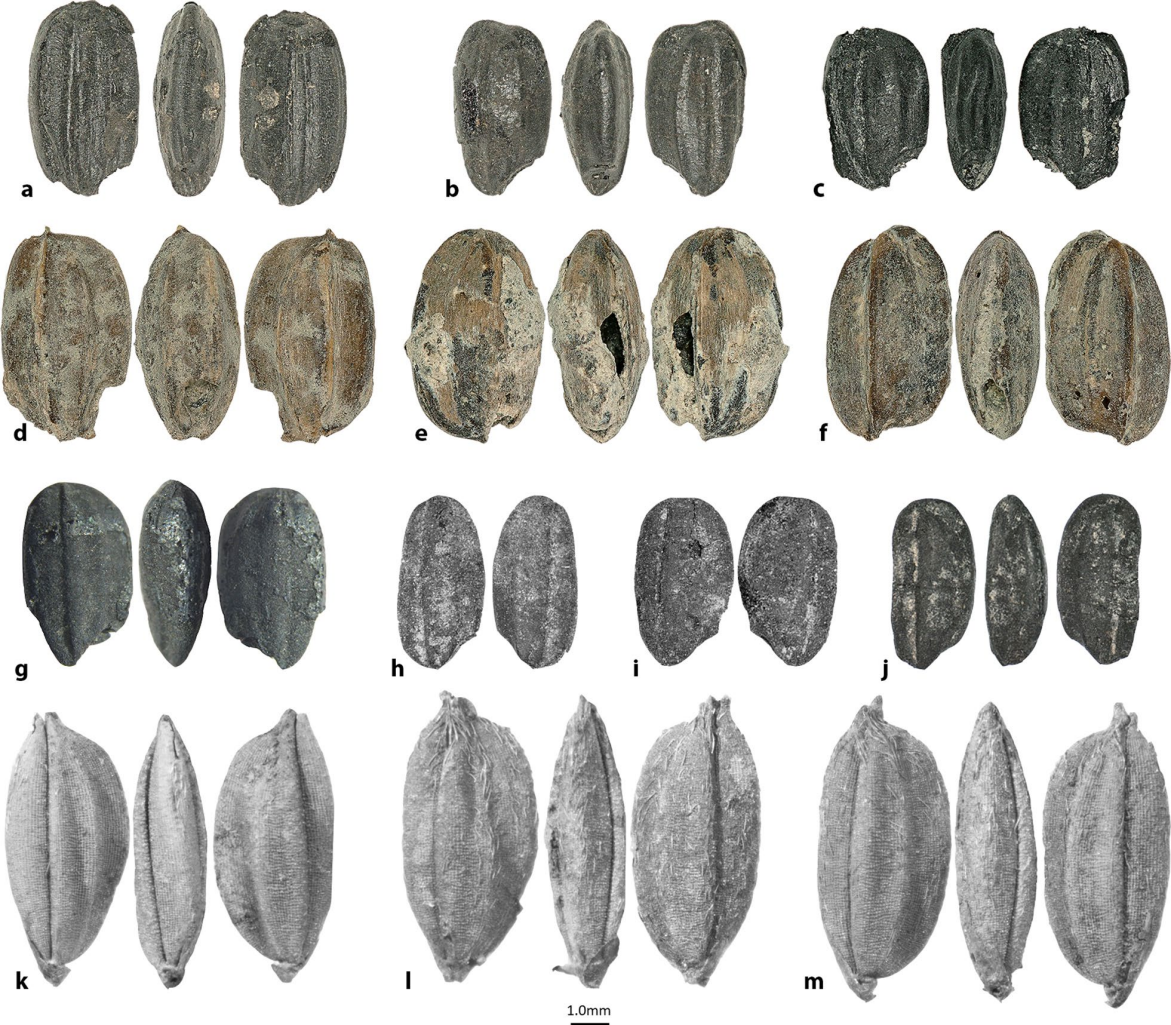
**a**, **b** three views of rice grains from Paykend (Mir-Makhamad et al. in review); **c** three views of a grain from Afrasiab; **d**–**f** three views of carbonized grains from Bukhara; **g** three views of a grain from Khalchayan (Chen et al. 2020); **h**, **i** two views of rice from Barikot (Spengler et al. 2020); **j** three views of a grain from Barikot (Spengler et al. 2020); **k**–**m** three views of three grains from the Astana Cemetery (Chen et al. 2012)

Today, rice is grown in parts of the Zerafshan Valley in Uzbekistan and in some particularly humid river valleys as far north as the southeastern corner of Kazakhstan. Recently, early rice grains were reported by Chen et al. (2020) from the site of Khalchayan. Two well-preserved grains (Fig. 2g) were recovered from the site, located along the Surkhan River of southeastern Uzbekistan. These two grains came from an ash pit located in an architectural complex that the excavators refer to as a dynastic shrine (Chen et al. 2020). The complex contained numerous artifacts that clearly express its elite standing, including coins, ceramic sculptures, and wall paintings. The two grains were directly dated to cal AD 230-416. The Surkandarya Region is a rich agricultural zone today and was likely far more prosperous in the past when glacial melt streams fed into the main artery of the Surkhan River throughout the summer. There is a published claim from a Soviet excavation in the 1970s that a large quantity of rice grains was recovered from one site in a cluster of sites alled the Nos 28, 29, and 61 group (Fig. 1) in Ferghana, dating to the early first millennium AD (Gorbunova 1986). Rice grains were also reported to have been recovered from mudbrick fragments at Munchak Tepe (fifth to seventh century AD) (Gorbunova 1986) in the Osh Region of Kyrgyzstan near the modern town of Kerkidon (Brykina 1974).

Desiccated grains of rice dating to the second half of the first millennium AD have also been reported from the Astana Cemetery in the Turpan region of Xinjiang (Chen et al. 2012; Fig. 2k–m). All of the grains from the Astana burial had their husks articulated and were located in the bottom of a ceramic pot, mixed with broomcorn and foxtail millet (*Setaria italica*) and bread wheat, as well as a few seeds of *Echinochloa crus-gali, Vaccaria segetalis, Seteria viridis,* and *Acroptilon repens*. Archaeological assessment of the mortuary goods left scholars uncertain as to the social status of the individual, but they estimated that the tomb dated between AD 327-460 (Li and Zhang 2006). Bashtannik (2008) reported recovering two ancient rice grains, one from the fourth to fifth centuries AD at Karaspan-tobe and another from the seventh century AD at Djuvan-tobe. Both medieval village sites are located along a tributary of the Syr Darya on the arid southern rim of Kazakhstan. While Bashtannik’s (2008) study was small, he did identify hundreds of grains of wheat, barley (*Hordeum vulgare*), and broomcorn millet (*Panicum miliaceum*), possibly suggesting that rice was still of minor significance in the early-medieval period. He also reported a small quantity of rice from the seventh century AD village of Konyr-tobe in the Otrar Oasis (Bashtannik 2008).

In the Semirech’ye region of southeastern Kazakhstan, a report of probable rice phytoliths in anthropogenic sediments was published from the sites of Tuzusai and Tseganka 8 (Rosen et al. 2000). The purported rice remains date to the mid- to late first millennium BC (Chang et al. 2002, 2003). Three seasons of additional archaeobotanical investigation at Tuzusai have failed to identify any macrobotanical remains of rice, calling into question the early claims (Spengler et al. 2017). Fan-shaped rice-like phytoliths were also reported from within sheep and goat dung from a desiccated 400 BC tomb in Xinjiang (Ghosh et al. 2008). The unlikeliness of rice at these high latitudes and in arid regions, especially at this early period means that further research is needed either confirming or rejecting these reports, as with the above-mentioned early phytoliths from East and South Asia.

**Fig. 3:**
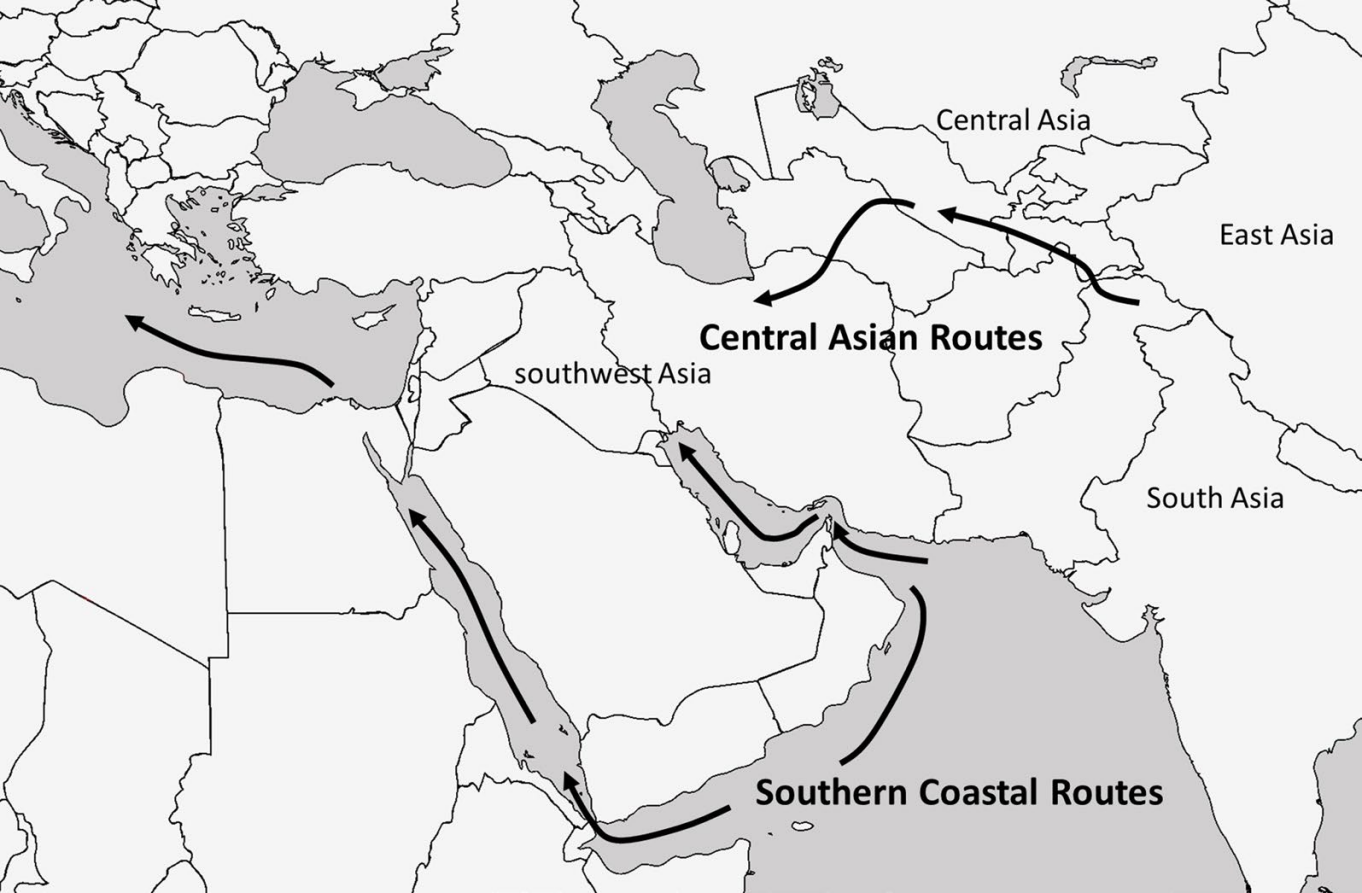
A map of Eurasia showing the two general routes of rice dispersal and the regions of Asia

Other slightly later claims of archaeobotanical rice in Central Asia include a mention in an early Soviet excavation at Teshik Kala, Uzbekistan, in the Khorasam Oasis dating to the seventh or eighth century AD (Brite et al. 2017) and at Merv, Turkmenistan, from a Sasanian phase, slightly earlier than Teshik Kala (Usmanova 1963). The report of grains from Merv noted that they came from the mudbrick and plaster floor, but further substantiation of this claim is warranted. Three grains of rice were also recovered from a hearth in a rabat at Paykend, in the Bukhara Oasis of Uzbekistan, dating to the Qarakhanid period at roughly AD 1100 (Mir-Makhamad et al. in review; Fig. 2a, b). There are thousands of millet and cereal grains in the Paykend assemblage, illustrating the continued rarity of rice through the medieval period. Ongoing research at both Qarakhanid capital cities of Afrasiab and Bukhara have identified rice grains; although, again, in both cases comprising a few grains among tens of thousands of identified grains and legumes. A midden deposit in the residential area of Afrasiab—ancient Samarkand—led to the recovery of three rice grains (Fig. 2c), and excavations in an area near the Ark at the heart of ancient Bukhara has led to the recovery of more than one hundred rice gains (Fig. 2d, e, f), among tens of thousands of other economic seeds (Fig. 3).

## South Asian Coastal Routes

### Historical Sources

The Greek writer, Diodorus Siculus’ *Bibliotheca Historica* (1967 [ca. 60 BC]: Book 2, Chapter 36), recapping accounts from now lost texts that supposedly dated several centuries earlier, mentioned rice being fed to soldiers of Eumenes of Cardia in 316 BC, presumably in the region of modern Iran. Additionally, Theophrastus (1916 [350-287 BC]: Book 4, Sect. 4) mentioned that rice was grown in India, a statement that indicates that he at least knew what rice was. It is likely that Theophrastus learned about the crop from members of Alexander’s expedition, and he specified that it was consumed as a gruel or mash. Although, Bertoni (2014) suggests that Theophrastus was not directly familiar with the plant or its geographic origins, noting that the first time he mentions rice, he associates it with Egyptian varieties of wheat. Rice is mentioned in several Greco-Roman texts, but all of their authors would have had the *Historia Planetarum* to build upon. Interestingly, Strabo (1924 [7 BC-AD 23]: Book XV, Volume 1: 18) is credited with stating that rice was grown in Susis/Susiana, likely based on lost earlier accounts of Aristobulus of Cassandria (Dueck 2015). Of an even earlier date, perhaps also related to rice cultivation in Susiana, are two texts from the Persepolis Fortification Archive, seemingly mentioning the handling of rice (Elamite *miriziš*) at Liduma (499 BC) and Kurra (587 BC)—both places were situated on the Royal Road between Susa and Persepolis (Hallock 1969; Tavernier 2007; Muthukumaran 2014). These accounts may further be supported by the *Shiji* as well (Sima 1993 [109-91 BC]: Book 123), claiming that rice was grown in Anxi, which is thought to be an old Chinese term for the Parthian Empire. According to Laufer, even the negative evidence for West Asian rice consumption in Chinese annals carries weight, “since the Chinese as a rice-eating nation were always anxious to ascertain whether rice was grown and consumed by foreign peoples” (Laufer and Sino-Iranica 1919: 372). While most historians recognize that rice was present in West Asia by the tail end of the first millennium BC, it was likely a commodity crop (Laufer and Sino-Iranica 1919). Muthukumaran (2014) suggests that there may be even earlier Akkadian references to rice on cuneiform tables, but we will not explore those here.

Dioscorides (2000 [AD 64]) also mentioned the use of rice for making bread, which he states is less nourishing than wheat. In the *Natural Histories,* Pliny the Elder discussed a drink like “barley water” made from rice (Stadler 1914), although he also was not familiar with what the rice plant looks like, or is possibly describing a different plant: “Rice leaves are fleshy, resembling leek but broader; the plant is 18 inches high, with a purple blossom and a root of a round shape like a precious stone” (1855 [AD 77–79]: Book 18, Chapter 13). Apicius, in his cookbook (1984 [first century AD]: Book 2, Chapter 2), claimed that rice was grown in West Asia; he specifically referred to it as a thickener for sauces. However, rice only appears in one percent of the 400 recipes in the book (Peterson 1980). Strabo (1924 [7 BC-AD 23]: Book 15, Chapter 1, Sect. 18), referencing earlier writings by Aristobulus, stated that “rice stands in enclosures of water, and it is included in beds”. It is independently reported by Strabo and the *Shiji* (Book 123:3163) in reference to the country Tiaozhi, 條枝, probably the kingdom of Charakene around modern Basra, in the southern reaches of the Euphrates-Tigris River basin (Hill 2009; Yang 2014). Strabo also stated that rice was grown in Babylonia, Bactria, Susa, and lower Syria (Stadler 1914). Other Greco-Roman sources ascribe medicinal properties to rice (*oryzae*), including Horace’s (2008 [35 BC]: Book 2, Chapter 3: 155) *Satires* and Celsus’ (1938 [first century AD]: Book 2, Chapter 20: 1) *De Medicina*. Celsus categorized the medicinal qualities of rice made into a gruel, with soft cheese, milk, nettle (*Urtica dioica*), mallow (*Malva* sp.), and raw eggs. Rice was mentioned in the early sixth century by the Byzantine physician, Anthimus (1996 [AD 500-525]) and by Galen (AD 150-210; Tassinari 2019), both of whom claim that rice porridge, made with goat milk, is good for an upset stomach. The medicinal use of rice to calm a stomach was widespread across the Mediterranean in the first millennium AD (Nesbitt et al. 2010). Classical references to rice as an Indian food, as a thickener for sauces, or as medicine, suggest that rice was a rare luxury ingredient in the Mediterranean during antiquity.

Rice was mentioned as a trade good in a logbook of Egyptian merchants on the Red Sea in the first century AD, in the *Periplus Maris Erythraei* (Schoff 1912). Both historical and archaeobotanical references to the grain at Red Sea ports in Egypt seem to suggest that it may have been an elite exchange good transported by nautical merchants. Other scholars have also emphasized the likelihood of rice being traded through the Red to the Mediterranean Sea as a commodity good. The weight of grain and the friction of overland travel may explain why it took so long to disperse across much of West Asia. As many scholars have pointed out, the cost of transporting grain over land in the ancient world was prohibitively expensive. Decker (2009) writes that rice became established as a local crop in Egypt by the second century AD. However, Cappers (2006) notes that there are only four reported references to rice out of 34,230 ancient papyrus texts of the third century BC to the eighth century AD. Early references to what is likely rice in the southern Levant appear in ancient Biblical translations by the first centuries AD, including the Samaritan Aramaic *Targum*, the Greek *Samariticon* and the Syriac *Peshitta* (Lӧw 1924; Feliks 1963; Rabin 1966). Judeans and Samaritans probably first encountered rice as a luxury import, and one scholar has proposed the Biblical ‘wheat of Minnith’ (Ezekiel 27:17), which refers to a luxury export from Judea to Tyre, was rice (Rabin 1966). Definitive references to rice (*orez*) as a local food crop appear in the *Mishna*, the classic second century AD rabbinic text (reflecting realia of the first centuries BC and AD).

The rabbinic references suggest that rice was well known in the southern Levant by this time but was not long-established (Feliks 1963). The *Mishna* distinguishes between rice grown within and outside the Land of Israel (*Mishna*, Demai 2:1), while related rabbinic sources refer to red rice grown on the plain of Antioch as distinct from Israel’s white rice (*Tosefta*, Demai 2:1; *Jerusalem Talmud*, Demai 2:1 – summarized in Feliks 2008). Other references in rabbinic texts suggest that rice was: (1) cultivated as a summer crop in Roman Palestine; (2) exported to neighboring regions; (3) stored husked or de-husked; and (4) roasted, baked, or boiled (*Mishna*, Shevi’it 2:7, 10; Hallah 1:4, 3:7, 10, 4:3; Pe’ah 8:3—summarized in Feliks 1963, 2008). References in the *Jerusalem Talmud* (redacted late fourth to early fifth century AD) and the Rehov inscription, suggest that local cultivation continued in early Byzantine Palestine. As elsewhere, rice in ancient Palestine was grown in those regions in which year-round irrigation was possible, such as Paneas, the Sea of Galilee, the upper Jordan Valley, and the Bet Shean Valley (Feliks 1963; 2008; Amar 2000; Safrai 1994). References to rice in the *Babylonian Talmud*, compiled in Mesopotamia (redacted fifth to sixth centuries AD, with additional editing until the early seventh century) indicate cultivation in Sasanian Iraq. These and other historical sources attest to rice cultivation in different parts of the Sassanid and Byzantine empires of Late Antiquity before the Islamization of both (Decker 2009).

Historians have suggested that rice cultivation expanded in the early Islamic period (Ashtor 1975 [1969]; Watson 1983; Amar 2000). One early Islamic testimony for the cultivation of rice outside of Khuzestan (west of the southern Euphrates-Tigris basin) comes from the physician ʿAlī b. Sahl Rabbān al-Ṭabarī (not to be confused with the historian, see below) in his *Firdaws al-ḥikmah fī al-ṭibb* (finished in AD 850), stating: “I saw rice in Ṭabaristān, which was 40 years old” (al-Ṭabarī 1928: 375). There are, however, indications that the cultivation of rice gained importance in Ērānshahr (in the Iranian heartland of the Sasanian Empire) during the Late Sasanian period. Perhaps best known in this respect is rice mentioned as one of the crops on which the land tax was based following the tax reforms of Khusraw I – with a tax rate of five-sixths of a dirham per *jarīb* (ca. 1,600 square meters) of rice (al-Dīnawarī 1912: 72–73; al-Ṭabarī 1928: 962). In the region around Basra, cultivation of rice is attested for the period of the Arab expansion, ca. AD 630 (Ibn al-Faqīh 1885: 187). It is the often overlooked in Rashīd al-Dīn’s *Kitāb-i āthār wa aḥyāʾ* that rice was mentioned during the Late Sasanian Period in Iran by Khusraw I Anūshirwān (Rashīd al-Dīn 1989; Petrushevskii 1960). Additionally, at the Late Sasanian court, rice and rice products were not only served (al-Thaʿālibī 1900: 585), but also played a role in Nawrūz rituals around the eleventh century, as noted in the *Kitāb al-maḥāsin waʾl-aḍdād* (1898; Ehrlich 1930)—likely the late ninth century author, Abū al-Ḥusayn ʿAlī ibn Mahdī al-Kisrawī, who wrote a *Book on Feasts and Nawrūzes* (Ibn al-Nadīm 1871-1872).

In the late tenth century, al-Muqaddasi mentioned rice cultivation for export in the Bet Shean Valley, and at least one text suggests that exportation of rice from Palestine continued in the thirteenth century (Amar 2000). Meanwhile, rice cultivation apparently expanded in Egypt, and became the chief staple cereal in the Fayyum (Ashtor 1975 [1969] quoting al-Muqaddasī and Ibn Haukal). In Mamluk Palestine (mid-thirteenth to early sixteenth centuries), local rice production was taxed (Amar 2000:75). It remained a minor crop, in relation to wheat and barley, and its appearance in medieval Arabic cookbooks is usually as a component of luxury dishes (Ashtor 1975 [1969]). According to Amar (2000), rice production declined in Palestine during the Ottoman period. Indeed, in the eighteenth century elite Ottoman Court, rice was apparently an imported commodity, mostly arriving from Egypt and reserved for banquets (Grehan 2007). Rice appears only to have become a widespread dietary staple throughout the Levant during the twentieth century.

As we elaborate upon below, rice remains elusive in archaeobotanical studies from West Asia and Europe, but it is found scattered across this vast region by the first millennium AD. Persian, Roman, and early Islamic irrigation infrastructure apparently facilitated expansion of cultivation, but its cultivation appears to have remained restricted to wetter microregions. As a result, rice remained a luxury food in West Asia and the Mediterranean.

### Archaeobotanical Data

Archaeobotanically, rice is rare in Central and southwest Asia, often appearing as one or two grains among thousands of domesticated crop remains at sites dating to the medieval period and later. Rice grains were recovered from a Parthian (ca 250 BC—AD 230) context at Susa, Iran—the same ancient city noted by Strabo (1924 [7 BC-AD23]) for rice cultivation. Miller (1981) noted 373 carbonized seeds of a short-grained variety of rice on the floor of Level 3A at Ville Royale II, in association with a jar that was likely a storage container for the grains, possibly as an offering. As with many of the historical sources, the context of this discovery, in a palace, suggests that rice may have originally been an elite commodity in West Asia. Impressions of rice husks in mudbricks were reported at sites in the South Dez plain near Susa, dating between 25 BC and AD 250; if these impressions are accurately identified, they might suggest local cultivation rather than import, but scholars are hesitant to accept the claims (Nesbitt et al 2010; Miller 2011). Preserved carbonized grains of rice were found in low abundance at the second century AD (*n* = 33 grains) Roman trading port of Quseir al-Qadim in Egypt, and a few grains were recovered from the Roman trading centers of Berenike and Myos Hormos (Cappers 2006; van der Veen 2011; Fig. 1). The grains at Quseir al-Qadim were mainly found in contexts near the port and found in association with mung bean (*Vigna radiata*) and coconut shell (*Cocos nucifera*) (van der Veen and Morales 2015). At both Red Sea coastal sites, rice grains were found in later stratigraphic layers, but remain at low abundance through the Islamic period. Scholars have argued that rice was imported to the region during the Roman period but may have been locally cultivated on a small scale during the Islamic period (van der Veen and Morales 2015; van der Veen et al. 2018). Another recent discovery of archaeobotanical remains of rice further supports a southern coastal route of dispersal; a fortified elite residence at the site of Mleiha in the United Arab Emirates was partially destroyed by a fire in the third century AD (Dabrowski et al. 2021). Carbonized rice grains at the site attest to its preparation as food and the processing of the husk from the grain, but the excavators still consider it a rare crop overall. Rice was recovered from three different rooms in the complex, as well as in the courtyard, and many of the grains were recovered in adhered clusters.

Much further afield, five rice grains were found in association with black pepper (*Piper nigrum*) from a series of trash pits filled with domestic waste dating to the early second century AD, near the center of the colonia Aelia Mursa in Croatia (Reed and Leleković 2019). The floated sediments constituted 850 L and were rich in grains and legumes that were likely locally grown, with rice and peppers presumably representing rare imports. Additionally, 196 charred grains of rice were recovered from the Roman military encampment or fort of Novaesium along the Rhine River, from the early first century AD, near the modern city of Düsseldorf (Knörzer 1966, 1970). The architectural structure where the grains were recovered has been interpreted by the archaeologists as a military medical facility, supporting the argument that rice in the Roman world served a medicinal role. A single probable rice grain was also recovered from the late first millennium AD from among sacrificial offerings at the Temple of Isis and Magna Mater in the Roman city of Mogontiacum, the capital of Germania Superior, where Mainz is today (Zach 2002). The scattered archaeobotanical remains of rice in West Asia and Europe support the view that rice was an exotic import in the Roman Empire and that in addition to medicine, it was an elite commodity for special meals (Zaouali 2009; Marton 2014; van der Veen and Morales 2015).

## Intensification of Rice Cultivation

Farmers around the world have engaged in increasingly intensive rice cultivation practices, even in the face of some of the least arable ecological conditions. The intensification of an extremely water-demanding crop in the arid regions of West Asia is testament to the ingenuity of ancient populations and illustrates that decision-making is often driven by cultural factors, such as taste and cuisine. Standing-water rice cultivation in most areas of West Asia is extremely labor intensive, it can lead to increased soil salinity, and is often at high risk for crop failure. Cultivation often requires regular drainage and re-irrigation of plots to avoid salt accumulation (Samuel 2001). The practice of regular water circulation is ethnohistorically attested in Afghanistan and Syria, where rice fields were also kept small and left fallow for three-year periods (Samuel 2001). Archaeobotanical and early historical evidence suggests that rice cultivation in West Asia was restricted to particularly rich and well-watered ecotopes and specifically for elite consumption until the late medieval period. In fact, Bacon (1980) suggested that in the recent past only the richest members of society could afford to make their pilaf out of rice (as opposed to barley).

Rice gradually increased in culinary significance across South Asia. Several millennia before rice became important in West Asia, barley was the most abundant grain at Hetapatti, but at later sites on the Ganges Plain, rice is more abundant (Pokharia et al. 2017). By the late first millennium BC, at the site of Rajdhani, rice grains were 250 times more abundant than barley grains, seemingly illustrating a major shift in cultivation practices over time. Pokharia et al. (2017) point out that all other cultivated crops in the Rajdhani assemblage combined, represent only about four percent of the overall cultivated crops, with rice significantly dominating the assemblage. In most South Asian archaeobotanical assemblages, rice is only one of a number of different crops, but it likely played an important role as a summer crop in seasonal rotation systems.

The prominence of rice cultivation around the world today is tied into crop-rotation cycles. Arguably, crop-rotation cycles are referenced in Chinese texts as far back as *The Book of Songs* (770-476 BC; Guo 1982); although, texts from the Han Dynasty, such as *The Book of Sisheng*, suggest a cycle of winter wheat and short-season millet was prominent. By the mid-first millennium AD, it is clear that these farming systems had been significantly intensified, with regionally specific forms of rotation (Guo 1982). During the Han Dynasty, collective labor projects involving irrigation expanded across northern China and spread into arid western China (Zhang and Tian 2015; Li et al. 2017). The demographic shift that marks the onset of the Han Dynasty, is likely tied into crop-rotation cycles and the expansion of summer-irrigated fields (Hosner et al. 2016). Archaeobotanically, the increased prominence of wheat in northern China starts to show up as early as 1000 BC (Deng et al. 2020) but really takes over in the Han Dynasty, which coincides with the culinary shift toward grinding and the use of noodles and steamed buns (Bray 1984). While multicropping systems of winter wheat and summer millet were practiced as far back as the first century BC, attested in Fan Sheg-Chih’s agricultural manual (Shih 1959), a crop-rotation cycle utilizing rice was not possible before the development of the rapid-maturing varieties.

Prior to the Song Dynasty in China, most rice was likely cultivated as a summer crop near natural water sources or on easily flooded land. Due to the long cultivation season of these early rice varieties, only one annual crop rotation would have been feasible and rice would have occupied the most arable land. Scholars have suggested that the intensification of rice cultivation in East Asia was tied into the development of a variety of rice with a shorter growing season (Ding 1957; Chang 1976; Anderson 1988; Simoons 1990). The rapid-growing Champa rice spread into the Yangtze River from Fujian in the eleventh century; historical sources specifically claim that it was introduced by Emperor Chang-tsung in 1011 (Anderson 1988, 2014). Large-scale collective labor projects, including irrigation, dam and levee construction, and forest clearing supposedly occurred in the Song period, accompanying the introduction of double-crop systems in southern China (Anderson 2014). The resulting demographic increase in East Asia would mirror the agricultural demographic transition in the north, with wheat/millet rotation cycles starting in the Han Dynasty, or the East-Asian-wide demographic transition that resulted from the introduction of Green Revolution crops and male-sterile hybrid rice in the 1960s.

Laufer and Sino-Iranica (1919) believed that rice cultivation in West Asia was not a prominent activity until the beginning of the Islamic period (after the seventh century AD). Watson (1983: 17) echoed these views, claiming that in “the eastern part of the caliphate, where rice had been grown in ancient times, and the early centuries of Islam saw an extension of its cultivation”. He further argued (1983: 17) that “rice early came to be grown in the Islamic world almost wherever there was water enough to irrigate it”. Watson directly tied the importance of rice in West Asian cuisines to the Islamic Expansion (starting in AD 623), as a fixture of his Early Islamic agricultural revolution. While many scholars have disputed aspects of Watson’s claims (Johns 1984), other scholars have broken down his claims, noting that the archaeological and historical records are not clear and all corollary factors for the intensification of farming have not been explored—likely much but not all of Watson’s thesis is true (Decker 2009; Fuks et al. 2020; Kennedy 2011; Squatriti 2014). Archaeobotanical studies in southwest Asia from Islamic periods are lacking, due to a focus on earlier periods. However, Samuel (2001) reports plant remains from a few village sites along the upper and middle Euphrates River. She did not recover any rice from contexts dating between the eighth and tenth centuries, but she did report a small amount of carbonized grains and rachis bases from eleventh through fourteenth century layers.

Arab geographers, such as al-Muqaddasi (AD 945-991), noted government-sponsored irrigation projects, specifically in this case along the Kur River of the Fars Region of Iran. Al-Muqaddasi also claimed that the ancient city of Istakhr, on the Pulvar River, a tributary of the Kur, was surrounded by rice fields and fruit orchards (Sumner and Whitcomb 1999). Rashid-al-Din Hamadani (1247-1318), a political figure and historian during the Ilkhanate, described a variety of rice that was popular in India that resembles Basmati (Anderson 2014). In the *Memoirs of Babur*, the ruler noted that, as his armies moved through the hills of the Kafiristan region of Afghanistan, the Kafir people fled or were killed, and his troops seized large quantities of rice (Babur 1922 [1483-1530]: Volume 2, Chapter 14). Abu'l-Fazl ibn Mubarak, the vizier to the sixteenth century emperor, Akbar, recorded recipes for preparing rice (Fazl 1873-1907 [1597]: Volume 1, Chapter 49–55). The Arabic geographer, al-Muqaddasi (945-991) discussed dams and irrigation systems to water rice fields and orchards, built around the lower Kur River near ancient Istakhr in Iran. The Mughal Emperor, Nuruddin Muhammad Jahangir (1569–1627) references the cultivation of rice in the Samarkand region (Jahangir 1909–1914 [1569–1627]). As a final point, there are some references to more arid-tolerant ancient forms of rice; for example, a specific form of dry-land and salt-tolerant rice may have been introduced to the southern Arabian Peninsula from India along the Red Sea trade routes, possibly linked to historic cultivation of Hassawi Rice (Dabrowski et al. 2021).

## Culinary Shift

Fuller and Rowlands (2011) note that a long lasting culinary divide has bisected Asia, with baking and grinding cuisines to the west and steaming and boiling cuisines to the east. In their discussion of this divide they focus on the culinary role of grain crops as they dispersed across Asia. Notably, millets are used to produce savory porridges in East Asia and in at least a few ancient examples are transformed into hard unleavened breads in West Asia. Similarly, wheat transforms into steamed buns, noodles, and dumplings in East Asia, as opposed to the baked breads of West Asia. The westward dispersal of rice appears to have resulted in a similar transformation. While Classical European texts refer to rice as an exotic (e.g. Theophrastus 1916 [350-287 BC]), largely medicinal plant (e.g. Anthimus; Celsus; Galen; and Horace), in cases where culinary properties are discussed, they refer to the use of rice water or ground flour. Classical writers also discuss the use of rice to make drinks, likely fermented, and bread. Feliks (1963) identifies sweet dishes of rice mixed with fruits and wine in Talmudic sources of the fifth to seventh centuries. Other historical references are made to a jelly-like dish or sweet porridge and sweet cakes (Nesbitt et al. 2010).

Even in early Arabic references to the culinary uses of rice, authors do not discuss dishes similar to pilaf; rather, they talk about rice beer, baked breads or pastries made from rice flour, and sweetened porridge with milk. The culinary use of rice in Turkic and Arabic cuisines appears to be a recent cultural adoption, possibly accompanying the wider cultivation of rice over the past few hundred years. Rice pilaf may be a recent adaptation of a boiled or fried barley dish that loosely shared many of the same properties (Bacon 1980). The earliest of the early Arabic cookbooks, dating somewhere between AD 940 and 960, included 615 recipes pulled together by Sayyar al-Warraq (Nesbitt et al. 2010; Nasrallah 2007; Marton 2014). Only a few of these recipes involve rice, notably in the form of rice bread and fermented beer. Nesbitt et al. (2010) note that, of the four early Arabic cookbooks dating to the thirteenth century, the ones written later in time have more recipes for rice. They also note that many of the recipes were for porridges, sometimes with meat, but often with sugar or honey, and in many of the dishes small quantities of rice, sometimes as flour, are used as a thickener. Samuel (2001) also references historical texts from the ninth and tenth centuries that mention sweet rice and milk porridges, with fruit syrups, saffron, sugar, grapes, figs, dates, or honey, and instructions for fermenting the grains or baking with rice flour. As the culinary role of rice changed, its prominence in West Asian cuisine increased.

Another preserved Arabic cookbook, the *Kitab al-Tabikh*, written by Muhammad bin Hasan al-Baghdadi in 1226, references several dishes with rice (Perry 2017). One thirteenth century cookbook presents nine dishes that contain rice, mostly pilafs; however, overall, rice is a minor component, and the *Book of Scents and Flavors the Banqueter Favors* was clearly written for an elite palate (Perry 2017). Another of these cookbooks, the *Kitab al-Wusla* written by Ibn al-Adim of Aleppo, who lived in Gaza and Egypt, and wrote the book in the early thirteenth century, provides the first clear reference to a pilaf-like dish, with rice, garlic, meat, and chickpeas (Nesbitt et al. 2010; Zaouali 2009). Nasrallah (2018) recently translated another Egyptian cookbook, *Treasure Trove of Benefits and Varieties at the Table*, which date to the fourteenth century and has additional recipes for rice, illustrating a gradually increasing culinary importance. Watson (1983) compared the number of recipes with rice to the number of ways to produce pickles, noting that the author presents dozens of ways to make pickled vegetables. Several early Arabic geographers or travelers also mention rice; Ibn Battuta (1325-1354) mentions consuming rice bread while on his trip from Mecca to Medina. Ibn Fadlan (2012 [921]) repeatedly references a dish of rice and fish. Chang Chun (1888 [1228]) mentioned rice growing in the Amu Darya river basin, presumably along the modern borders of Uzbekistan and Turkmenistan. The number of references to savory rice dishes with meat increases over time. Several scholars have pointed out that many of the customs associated with making pilaf, such as washing the rice, are evident in these early cookbooks (Zaouali 2009; Nesbitt et al. 2010; Marton 2014). Rice became prominent in the wet river valleys across West Asia before the first European explorers reached the region, by which time pilaf-like dishes were already popular. For example, Burnes (1834: Book 1: 228) references a rice and meat dish in Bukhara. Mac Gahan (1876: 318) mentions eating pilaf while traveling along the Amu Darya; Marvin (1881) describes pilaf in Merv; Schuyler (1877: 125) describes it in Turkmenistan.

## Conclusions

The gradual transition from a wild to a domesticated population of rice in the lower Yangtze basin, as is archaeobotanically visible at the sites of Tianluoshan and Hemudu, culminated around 4600 BC. This first anthropogenic trait of domestication became fixed into a cultivated population in East Asia, and from there spread into South Asia, likely through rich mountain passes. The allele for tough rachises introgressed into South Asian rice representing domestication. The transfer of the allele from fully domesticated *japonica* rice to previously wild *indica* rice ultimately resulted in separate basmati and pearl rice clades. It is rather clear from the historical and archaeobotancial data that rice dispersed along two routes on its journey to the west. One of these routes crossed the deserts of southern Central and southwest Asia and the other traversed the coast through nautical routes from India and up the Red Sea to the Mediterranean.

Rice gradually increased in culinary significance across South Asia and eventually spread into Central and arid southwest Asia to become a prominent grain by the late medieval or early modern period. Much remains to be discovered regarding the precise timing of this westward dispersal and the extent to which greater irrigation and public-works projects were tied into the increased prominence of the crop through time. However, the earliest culinary role for rice in West Asia was in sweet porridges and as medicine; its savory staple role developed later. Archaeobotanical data in the southern Himalaya does not stretch back far enough, but the presence of rice cultivation in these rich river valleys supports the likelihood of a dispersal along this route. While rice is an important part of Central Asian cuisines, it probably did not become a significant crop west of the Indus region until well after the Islamic Expansion. The pilaf or other rice-based dish that exemplifies many Central and southwest Asian cuisines probably only originated over the past millennium.

## Data Availability

All discussed data is presented in the text of figures.
